# Electrophysiological signature of the interplay between habits and inhibition in response to smoking‐related cues in individuals with a smoking habit: An event‐related potential study

**DOI:** 10.1111/ejn.15942

**Published:** 2023-03-21

**Authors:** Julien Dampuré, Paola Agudelo‐Orjuela, Maartje van der Meij, David Belin, Horacio A. Barber

**Affiliations:** ^1^ Facultad de Psicología Universidad de La Sabana Chía Colombia; ^2^ Instituto Universitario de Neurociencia (IUNE) Universidad de La Laguna (ULL) Tenerife Spain; ^3^ Faculté de Psychologie Université Catholique de l'Ouest 79500 Niort France; ^4^ Universidad Externado de Colombia Bogotá Colombia; ^5^ Departamento de Psicología Cognitiva Universidad de La Laguna (ULL) Tenerife Spain; ^6^ Department of Psychology University of Cambridge Cambridge UK; ^7^ Basque Center on Cognition, Brain and Language (BCBL) Donostia‐San Sebastián Spain

**Keywords:** cue reactivity, EEG ERP, habits, inhibition, smoking habit

## Abstract

The rigid, stimulus‐bound nature of drug seeking that characterizes substance use disorder (SUD) has been related to a dysregulation of motivational and early attentional reflexive and inhibitory reflective systems. However, the mechanisms by which these systems are engaged by drug‐paired conditioned stimuli (CSs) when they promote the enactment of seeking habits in individuals with a SUD have not been elucidated. The present study aimed behaviourally and electrophysiologically to characterize the nature of the interaction between the reflexive and reflective systems recruited by CSs in individuals with a smoking habit. We measured the behavioural performance and associated event‐related potentials (ERPs) of 20 individuals with a smoking habit and 20 controls, who never smoked regularly, in a modified Go/NoGo task during which smoking‐related CSs, appetitive and neutral pictures, presented either in first or third‐person visual perspective were displayed 250 ms before the Go/NoGo cue. We show that smoking‐related cues selectively influence early incentive motivation‐related attention bias (N2 after picture onset), motor readiness and behavioural inhibition (Go‐P3, NoGo‐P3 and Pc) of individuals with a smoking habit only when presented from a first‐person visual perspective. These data together identify the neural signature of the aberrant engagement of the reflexive and reflective systems during the recruitment of an incentive habit by CSs presented as if they had been response‐produced, that is, as conditioned reinforcers.

## Graphical abstract

Using a new validated picture library comprising neutral, appetitive and smoking‐related pictures presented in first‐ or third‐person visual perspective (1‐VP or 3‐VP, respectively), Dampuré et al. showed that individuals with a smoking habit evoke a different late P3 in response to smoking‐related cues than controls, but only when the pictures are in 1‐VP. Smoking‐related pictures also influenced the motor response‐locked Pc in individuals with a smoking habit more than appetitive or neutral pictures. Together these results suggest that when presented as if they were response produced, for example, as conditioned reinforcers, drug‐paired conditioned stimuli facilitate the engagement of habitual responding in smokers.

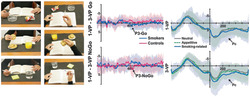

## 1 INTRODUCTION

Substance use disorder (SUD) is a chronic relapsing disorder characterized by an aberrant motivation for, and the compulsive seeking and taking of, drugs (APA, 2013). The transition from controlled, recreational drug use to the rigid engagement in drug seeking behaviour that persists despite adverse consequences, the hallmark feature of SUD, has been suggested to result from the development of maladaptive drug seeking habits (Belin et al., 2013; Belin & Everitt, 2010; Fouyssac et al., 2022; Zilverstand et al., 2018).

At the neural systems level, this progressive functional recruitment of the habit system over the course of a history of drug use is reflected by a shift in the corticostriatal systems mediating the influence of drug‐paired conditioned stimuli (CSs), on behaviour from the ventral to the dorsolateral striatum (Belin et al., 2013; Cox et al., 2017; Everitt & Robbins, 2016; Vollstadt‐Klein et al., 2010). While the motivational influence of CSs on behaviour is associated with the activation of the nucleus accumbens in recreational users, the same CSs functionally engage the dorsal striatum‐dependent habit system in individuals with a long history of cocaine, alcohol, heroin or nicotine use (Cox et al., 2017; Detandt et al., 2017; McClernon et al., 2009; Volkow et al., 2006; Vollstadt‐Klein et al., 2010; Xie et al., 2014).

Activation of the habit system in cue‐provoked craving tasks has been shown to be one of the best predictors of actual long‐term relapse (Zilverstand et al., 2018), otherwise strongly associated with the intensity of craving itself (Vafaie & Kober, 2022). Thereby this observation suggests that in individuals with SUD, drug‐paired CSs not only engage overt motivational states but they also recruit covert mechanisms through their influence on a bottom‐up reflexive system, which, in conjunction with an inherent impaired top‐down control system, biases attention towards drug‐related stimuli (attention bias) and engages drug seeking habits and foraging behaviour (Approach bias) (Bechara et al., 2006; Belin et al., 2013; Donamayor et al., 2021; Field & Cox, 2008; Luscher et al., 2020; Ramey & Regier, 2019; Watson et al., 2012). Attention bias has therefore been suggested to reflect an implicit component of craving (Tiffany & Wray, 2012), predictive of relapse and treatment efficacy (Goldstein & Volkow, 2011; McKay, 1999; Tiffany et al., 2012), that is behaviourally and electroencephalographically (EEG) characterized by faster response times and by modulations of early (e.g., posterior N1 and N2) and late (e.g., Late Positive Potential LPP, 400–600 ms) event‐related components, respectively (Detandt et al., 2017; Littel & Franken, 2007; Minnix et al., 2013; Rangaswamy & Porjesz, 2014; Robinson et al., 2016; Wiers et al., 2013).

The loss of top‐down cognitive control over these covert mechanisms, which contributes to the stimulus‐bound and compulsive nature of the pursuit of the drug (Belin et al., 2013; Everitt et al., 2008; Everitt & Robbins, 2005), has been related to alterations of the reflective system, which comprises the dorsolateral prefrontal (DLPFC) and the anterior cingulate (ACC) cortices (Zilverstand et al., 2018). Weakening of early inhibitory control over prepotent responses exerted by these prefrontal cortical regions has been characterized in experimental tasks such as the Go/NoGo, the Stop‐signal or the Stroop task as two inhibitory‐specific event‐related potential (ERP) components: the anterior N2 and the P3 (Kok et al., 2004). The anterior N2, a negative‐going wave with a peak occurring at frontocentral regions approximatively 200–300 ms after stimulus onset, is associated with the conflict monitoring function of the ACC (Mathalon et al., 2003, 2003; Pandey et al., 2012; van Veen & Carter, 2002). The P3, which culminates at frontocentral or parietal regions around 300 and 500 ms after stimulus onset, has been related to the active motor inhibition process per se (Waller et al., 2021). Accordingly, individuals with a smoking habit, referred to as smokers henceforth, tend to evoke an anterior N2 and P3 of smaller amplitude than controls, in particular in the NoGo condition (Luijten et al., 2011; see Pandey et al., 2012, for similar results with patients with an alcohol use disorder). Modified Go/NoGo tasks in which presentation of a drug‐paired CS before a Go/NoGo signal or as the Go/NoGo signal itself enables the investigation of the interactions between the reflexive and reflective systems have helped reveal that individuals with alcohol, opiate or cocaine use disorder show higher rates of false alarms and smaller N2‐NoGo and P3‐NoGo amplitudes (Blanco‐Ramos et al., 2019; Campanella et al., 2020; Detandt et al., 2017; Rangaswamy & Porjesz, 2014). A smaller P3‐NoGo, which appears to be a more reliable characteristic of SUD than the anterior N2, may therefore be a valuable neuromarker of the inhibitory deficits that predict relapse (Cohen et al., 1997; Fallgatter et al., 1998; Kamarajan et al., 2005; Colrain et al., 2011; Luijten et al., 2014; for review, see Rangaswamy & Porjesz, 2014).

Together these observations suggest that the engagement of the reflexive system by cocaine‐, opioid‐ or alcohol‐paired CSs challenges and exacerbates the weakness of the reflective system in individuals with SUD, thereby facilitating the expression of covert rigid stimulus‐bound behaviours. However, it remains to be established if it is also the case for nicotine addiction, in which the nature of the interaction between the reflexive and reflective systems seems to differ from that of other SUDs. Indeed, while some studies have reported larger P3‐Go/NoGo following the presentation of drug‐related CSs as compared with neutral stimuli (Detandt et al., 2017), others have provided behavioural and/or neurophysiological evidence of a general deficit of the reflective system that is independent of CSs recruitment of the reflexive system, for example, irrespective of whether a CS is presented before the Go/NoGo cue or as the Go/NoGo cue (Buzzell et al., 2014; Evans et al., 2009; Liu et al., 2019; Luijten et al., 2011; Yin et al., 2016).

Therefore, in the present study, we sought (i) to characterize the nature of the interactions between reflexive and reflective processes in response to drug‐paired CSs in individuals with a smoking habit. For that, ERPs were measured in twenty individuals with an engrained smoking habit (smokers) and twenty control individuals who had no history of regular smoking (controls) during a modified Go/NoGo task in which pictures of neutral, smoking‐related and appetitive stimuli were displayed 250 ms before a Go/NoGo trigger cue. The latter were included in order to discriminate the specific effect of drug‐paired CSs from that of general appetitive arousal on ERPs (Versace et al., 2017).

Drug‐paired CSs are not just passively experienced, but instead, they are often response‐produced, acting as conditioned reinforcers, which support foraging behaviour over delays to reinforcement and contribute to the development of incentive habits (Belin et al., 2013; Belin & Everitt, 2010; Fouyssac et al., 2022; Olausson et al., 2004). Therefore, CSs are often experienced from a first‐person perspective by individuals with SUD actively engaged in their drug seeking habits, thereby fostering the integration of addiction‐specific multisensorial representations (Yalachkov et al., 2012). Thus, we also sought (ii) to characterize the influence of the visual perspective of the CSs (first‐ vs. third‐person), which differentially recruits automatic embodiment (Canizales et al., 2013) and sensorimotor activation (Canizales et al., 2013; Galang et al., 2020), on the interactions between the reflexive and reflective systems. For this, we created a new library of images that was validated on an independent cohort of individuals.

Finally, beyond cue reactivity, the stimulus‐bound response tendency characteristic of individuals with a smoking habit should also be reflected by modulations of the response‐locked potentials evoked in the Go condition, that is, when no inhibition is required (Donkers & van Boxtel, 2004). Surprisingly, these response‐locked potentials, especially those evoked in correct Go trials, have not been thoroughly studied in SUD (Luijten et al., 2014, 2016). We sought (iii) to characterize in smokers the nature of the two main Go response‐associated ERP components, namely, the correct response negativity (CRN) and the correct positivity (Pc), representing an early (peak 80 ms after the response) and late (peak 300 ms after the response) components, respectively (Somon et al., 2019). The Pc, a P300‐like component related to the monitoring of the performance based on the representation of the outcome (i.e., subjective emotional significance, see Falkenstein et al., 2000), but not the CRN, which falls in the error‐related negativity (ERN) time window likely related to the evaluation of the accuracy of the response produced compared with the expected response, is reduced when two stimuli call for a similar motor‐response (Mathalon et al., 2002). Thus, we tested the hypothesis that the Pc is reduced selectively smokers when they show response facilitation brought about by the presentation of smoking‐related CSs in the Go condition, revealing the behavioural and neurophysiological signature of a drug‐related habit.

We first anticipated that appetitive and smoking‐related stimuli would evoke a more positive LPP than neutral pictures in both groups. We then expected that smoking‐related CSs would recruit early attentional processes selectively in smokers, reflected as a decrease of the posterior N1/N2 amplitude, followed by the manifestation of motivation‐related processes reflected by an increase of the late positive potential amplitude (i.e., LPP) (Littel & Franken, 2007; Minnix et al., 2013; Rangaswamy & Porjesz, 2014; Robinson et al., 2016). Since motivation‐related processes occur about 300 ms after stimulus onset (Robinson et al., 2016), the motivational component of the response to the stimulus presentation (i.e., the LPP) was expected to be detected within the N2/P3‐Go/NoGo time window (Agudelo‐Orjuela et al., 2021).

According to the interactive reflexive–reflective system model, we expected an exacerbation of the difference between smokers and controls in the amplitude of the N2‐Go/NoGo and/or the P3‐Go/NoGo elicited by the presentation of smoking‐related as compared with neutral and appetitive stimuli (Detandt et al., 2017). Similarly, we expected the reflective system of smokers to be even more challenged by the activation of the reflexive system by drug‐paired CSs presented as first‐person (1‐VP) compared with third‐person visual perspective (3‐VP), reflected at the neurophysiological level by an increase of the P3‐NoGo amplitude (Detandt et al., 2017). Finally, in these individuals with a smoking habit, we also expected that the reliance on stimulus–response association would facilitate response processing, as reflected by a decrease of the P3‐Go amplitude, when both the drug‐ and task‐related cues converge towards a similar response (Detandt et al., 2017; Rose et al., 2001; Watson et al., 2012; Wiers et al., 2013).

## 2 MATERIALS AND METHODS

### 2.1 Participants

Twenty‐three individuals with a smoking habit and twenty individuals who never regularly smoked (fifteen women in each group) voluntarily participated in this study, having signed an informed consent before the session. All participants were Spanish from Tenerife (Spain) and were recruited by convenience in the towns of La Laguna and Santa Cruz de Tenerife. They received 15€ after the completion of the study, which was approved by the Ethical Committee of the University of La Laguna (Tenerife, Spain).

Each participant first filled out an online questionnaire in which demographic and personal data were collected (gender, age and smoking history) and neuropsychological tests (Barratt Impulsiveness Scale; STAI‐R; Beck's Depression Questionnaire; and the Positive Affect and Negative Affect Scale, PANAS) (Salvo & Castro, 2013; Sandin et al., 1996; Sanz et al., 2005; Watson et al., 1988) were self‐administrated online using the Psytoolkit online software (Stoet, 2010). Smokers were also assessed for their smoking history (i.e., number of years of active smoking and number of cigarettes smoked per day) and their score in both the Spanish adaptation of the Fageström test (Becona & Vazquez, 1998) and the Obsessive–Compulsive Smoking Scale (Hitsman et al., 2010) in order to establish the severity of their smoking habit (see Table 2).

Before the beginning of the experimental session, each participant was subjected to a measurement of their level of carbon monoxide (C.O.) using a Smokerlyzer (Bedfort Scientific Ltd., Rochester, UK). Then, each participant filled out the STAI‐E and was given a short clinical interview led by J.D. in order to record any current medication and to detect any potential psychiatric, including other substance use, disorders using the MULTICAGE CAD‐4 questionnaire (Pérez et al., 2007). Any participant (control or smoker) who scored strictly above 2 (out of 4) in at least one of the eight subscales of the MULTICAGE CAD‐4 (alcohol, illegal drugs, pathological gambling, Internet, video games, compulsive spending, eating disorders and sex addiction) was automatically excluded from the study. Three smokers were excluded before completion of the study due to a demyelinating disease, ongoing antidepressant (Xeristar 60 mg) medication and suspicion of an alcohol use disorder, so that the final sample size was *n* = 20 per group. Finally, the spontaneous level of craving of the smokers was measured before and after the experimental session on a 31‐point scale.

### 2.2 Apparatus

Stimuli were presented using the E‐Prime 2.0 software (PST) on a 17‐in. monitor screen at a 768 × 1024 pixels resolution and 100 Hz controlled by a P.C. The EEG signal was collected using the EasyCap system (BrainVision) equipped with 27 Ag/AgCl electrodes arranged in the international 10–20 system and referenced to the left mastoid. Four additional electrodes were used in order to provide bipolar recordings of the horizontal and vertical electrooculogram, two located at the outer canthus of each eye and two at the infraorbital and supraorbital regions of the right eye. The electrical activity was recorded and amplified with a bandwidth of 0.01–100 Hz and sampled at 500 Hz with impedances kept below 5 kΩ (electrooculogram <10 kΩ).

Gaze position was also measured using an EyeLink 1000 system (S.R. Research Ltd., Ontario, Canada) at a 1000 Hz sampling rate, and synchronized to the E‐Prime software. During the session, the participant sat at a distance of 70 cm from the screen, with the head resting on a chinrest adjusted to a comfortable position. A 7‐point calibration was performed just before the initiation of the experiment.

### 2.3 Materials

**FIGURE 1:**
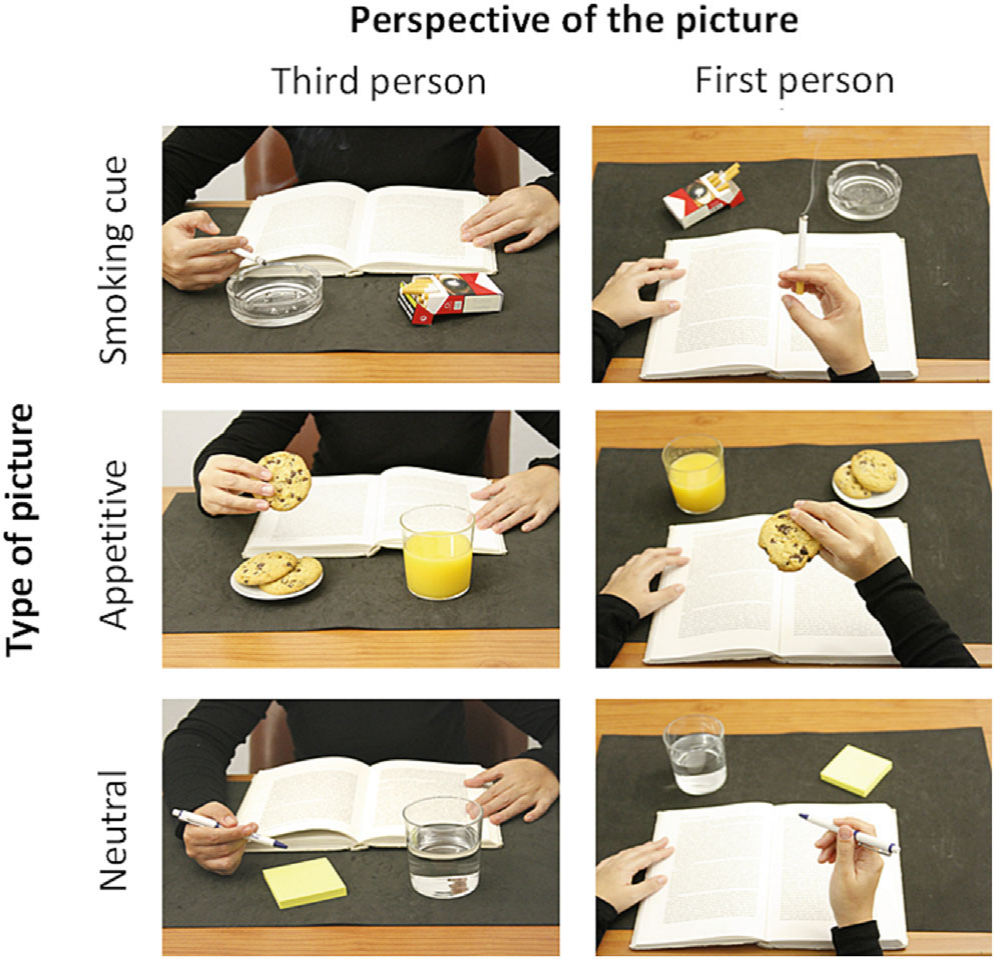
Example of smoking‐related, appetitive or neutral pictures presented either from a first‐ or third‐person visual perspective.

The 240 pictures created for this study were taken by a member of the research team with a Canon professional camera in a room without windows, ensuring consistent luminosity. Each photo depicted a person interacting with one of three objects placed on a desk. Objects were chosen in order to create three sets of 80 pictures: smoking‐related pictures displaying smoking‐related objects (e.g., burning cigarette, lighter, cigarette pack etc.), appetitive pictures (e.g., muffin, cookie, chocolate etc.) and neutral pictures displaying office items and stationery (e.g., pen, book, paper sheets etc.). Half the 80 pictures of each set were taken in 3‐VP (i.e., facing the actor), the other half being taken in 1‐VP. Examples of each stimulus are presented in Figure 1. Each photo was then post‐processed using ImageJ (NIH) in order to homogenize size and luminance. An ANOVA performed on the intensity with the Picture type (appetitive, smoking‐related, neutral) and the Visual perspective (first‐person, third‐person) as between‐subject factors confirmed no differences between picture types (*F*_2,114_ = 2.04, *p* = 0.14) or visual perspectives (*F*_1,114_ = 0.03, *p* = 0.95). The emotional valence and arousal‐inducing properties of each picture were assessed and validated online on an independent pool of 83 participants (76% females, 15% of smokers) using the Psytoolkit software (Stoet, 2010).

### 2.4 Task paradigm and procedure

The GO/NoGO task lasted approximately 30 min. A trial began with a grey screen for 450 ms, immediately followed by a 250 ms presentation of a picture (smoking‐related, appetitive or neutral) displayed at the centre of the screen on a grey background. The frame of the picture was then coloured for 450 ms in blue or green (counterbalanced between subjects), indicating a Go or a NoGo trial, after which the picture was replaced by a grey screen for 950 ms. Participants were instructed to respond in the Go trials by pressing the space bar of the keyboard with the right index as quickly and accurately as possible and to withhold that response in the NoGo trials. They were also told not to look directly at the border of the picture. Gaze position followed by an Eyelink 1000 (S.R. Research) was synchronized with the E‐Prime software. If a participant's gaze was detected to be directed at the picture frame the trial was immediately interrupted, and an error message appeared on the screen. The cancelled trial was then presented again later in the experimental sequence.

There were 120 trials for each type of picture (80 in the Go condition and 40 in the NoGo condition) presented in random order in blocks (smoking‐related, appetitive or neutral), the order of which was counterbalanced between participants. Participants were given the opportunity to rest for up to 5 min between each block.

### 2.5 EEG preprocessing

Data were processed offline using the Brain Analyzer software (Brain Products GmbH, Gilching, Germany). The EEG recorded data were filtered with a 0.1–30 Hz band‐pass, re‐referenced to the algebraic mean of the activity at the two mastoids and corrected for ocular artefact using independent component analysis (ICA, Makeig et al., 1995; Jung et al., 1998). Raw data, only including correct responses, were then segmented in 2‐s epochs centred on the time of picture onset. Manual artefact rejection was carried out, resulting in a similar trial rejection rate between smokers and controls (3.5% and 3.2%, respectively, *t*_38_ = 0.30, *p* = 0.77). Data were then subjected to further processing in order to extract the relevant electrophysiological data, with three objective‐driven segmentations carried out as per the following pipeline:

1. The 200 ms period preceding stimulus or response onset was used to calculate the baseline;
2. Averages per participant were computed for each segmentation, picture type and Go/NoGo stimulus.
3. Grand averages were then calculated and a detection of the maximal peak amplitude was performed, informed by the time windows identified in the literature;
4. The mean amplitudes (see selected time‐windows below for each component) around the peak were exported and analysed.

This pipeline led to the extraction of event‐ or response‐related potentials that were locked on:

1. Picture onset, reflecting the reflexive system‐related N2 (240–300 ms), an ERP component with a posterior distribution that captures early motivation‐related attentional mechanisms.
2. The Go/NoGo cue onset in order to isolate the reflective system‐related N2‐Go/NoGo (250–300 ms) and the P3‐Go/NoGo (310–370 and 420–470 ms), two ERP components with an anterior distribution.
3. The correct responses in the Go condition to isolate the post‐response potential Pc (220–300 ms), a RRP with a central distribution.

### 2.6 Statistical analyses

Data are presented as means ± 1 SEM or box plots (medians ± 25% and min/max as whiskers) and were analysed using “R” software (version 3.4.0) with the ULLRToolbox (https://sites.google.com/site/ullrtoolbox/home). Assumptions of normality and homogeneity of variance were verified using the Shapiro–Wilk's and Cochran test, respectively.

The emotional valence and arousing properties of each picture of the bespoke first‐ and third‐person visual perspective‐matched picture library created for the purpose of the study was established in an independent cohort of 83 individuals, including 12 smokers, using an analysis of variance (ANOVA) with smoking status (smokers vs. non‐smokers) as between‐subject factor and the visual perspective (1‐VP vs. 3‐VP) and picture category (appetitive, smoking‐related and neutral) as within‐subject factors.

Repeated‐measure ANOVAs were used to compare response times, ERPs or RRPs amplitudes with Group (smokers vs. controls) as between‐subject factor, and the type of cue (Go vs. NoGo), the type of picture (smoking‐related, appetitive or neutral), the visual perspective (1‐VP vs. 3‐VP), the Regions (Frontal‐left, Frontal‐right, Frontocentral, Central‐left, Central‐right and Central, Parietal) and the Electrodes (e1, e2 and e3) as within‐subject factors.

The efficiency of the inhibitory mechanism in the Go/NoGo task was evaluated by the error rate and the response time (in milliseconds) of the participants. Response time was defined as the time that elapsed between the appearance of the Go cue and the response (key press). Response times were first submitted to a log‐transformation and then subjected to an ANOVA using the Group (smokers vs. controls) as between‐participant factor, and the type of picture (smoking‐related, appetitive or neutral) and the visual perspective (1‐VP vs. 3‐VP) as within‐subject factors. Finally, false alarms and misses were analysed using chi‐squared tests (χ^2^).

Upon confirmation of significant main effects, differences among individual means were analysed using the Newman–Keuls post hoc test or planned comparisons.

For all analyses, significance was set at α = 0.05 and effect sizes were reported as partial eta squared (_p_η^2^) for every statistically significant effect.

## 3 RESULTS

### 3.1 Validation of the image bank

The administration of our new first‐ and third person visual perspective‐matched picture library to an independent cohort of 83 individuals, including 12 smokers, confirmed that the three picture categories were considered to differ in their emotional valence and arousing properties (main effect of picture: *F*_2,162_ = 131, *p* < 0.0001, _p_η^2^ = 0.76 and *F*_2,162_ = 8.43, *p* < 0.0005, _p_η^2^ = 0.24, respectively) (Table 1). Pictures of appetitive stimuli were deemed more appetitive and more arousing than those of neutral (*t*_405_ = 4.49, *p* < 0.0001 and *t*_405_ = 4.39, *p* < 0.0001, respectively) and smoking‐related stimuli (*t*_405_ = 12.36, *p* < 0.0001 and *t*_405_ = 0.36, *p* = 0.72), respectively. In contrast, pictures of smoking‐related stimuli were deemed more arousing (*t*_405_ = 4.03, *p* = 0.0001) but less appetitive than pictures depicting neutral stimuli (*t*_405_ = 7.86, *p* < 0.0001). The visual perspective did not influence the emotional properties of the image (*F*_1,82_ = 2.89, *p* = 0.09, *F*_1,82_ = 1.74, *p* = 0.19 for emotional valence and arousal property, respectively). Smokers differed from non‐smokers in their judgement of the emotional valence, but not of the arousing properties of the appetite and smoking‐related pictures (main effect of picture: *F*_2,162_ = 9.94, *p* < 0.001, _p_η^2^ = 0.20 and *F*_2,162_ = 8.43, *p* < 0.001, _p_η^2^ = 0.09, respectively) (Table 1). Planned comparisons revealed that while smokers did not differ from non‐smokers in their rating of the emotional valence of neutral pictures (*t*_265_ = 0.18, *p* = 0.86), they judged smoking‐related pictures more appetitive (*t*_265_ = 5.63, *p* < 0.0001) and appetitive pictures less appetitive (*t*_265_ = 2.33, *p* = 0.02).

**TABLE 1:** Outcome of the evaluation (mean ± SD) of the emotional valence and arousal property of pictures as a function of their type (appetitive, smoking‐related or neutral), the visual perspective in which they were taken (from a first‐ or third‐person perspective) and the status of the participant (smokers or non‐smoker controls).

Type of picture	Visual perspective	Valence		Arousal	
		(from 1 to 7)		(from 1 to 7)	
		Controls	Smokers	Controls	Smokers
Appetitive	First‐person	5.4 ± 1.2	4.3 ± 1.5	4.3 ± 1.5	4.3 ± 1.5
	Third‐person	5.5 ± 1.2	4.3 ± 1.5	4.3 ± 1.5	4.3 ± 1.5
Smoking‐related	First‐person	2.6 ± 1.4	4.1 ± 1.8	4.1 ± 1.8	4.1 ± 1.8
	Third‐person	2.5 ± 1.3	4.0 ± 1.8	4.0 ± 1.8	4.0 ± 1.8
Neutral	First‐person	4.4 ± 1.1	3.5 ± 1.3	3.5 ± 1.3	3.5 ± 1.3
	Third‐person	4.6 ± 1.3	3.5 ± 1.3	3.5 ± 1.3	3.5 ± 1.3

### 3.2 Characteristics of the experimental groups

Smokers, who had a much higher level of carbon monoxide than control individuals (14.05 ± 9.32 ppm vs. 4.75 ± 1.45 ppm) (*F*_1,38_ = 18.76, *p* < 0.0001, _p_η^2^ = 0.33), were moderate smokers as determined by their scores in the Fagerström test and the OCSS (Schane et al., 2010; Wilson et al., 1999) (Table **2**). Smokers showed a characteristic increase in their spontaneous level of craving following the experimental session (16.2 ± 10.9 vs. 18.42 ± 10.3, *F*_1,19_ = 6.23, *p* = 0.02, _p_η^2^ = 0.25), and as expected (Bickel et al., 1999; Mitchell, 1999; Potvin et al., 2015), they were more impulsive than controls, as revealed by their score in the BIS (F_1,38_ = 11.19, *p* < 0.002, _p_η^2^= 0.23). However, smokers did not differ from controls in their levels of anxiety, mood or depression, as assessed by the STAI, the PANAS and the Beck questionnaire, respectively (Table 2).

**TABLE 2:** Demographic and personal data of smokers and controls.

	Smokers	Controls	*T* value	*p* value
Age	30.8 ± 12.4	30 ± 12.3	0.22	.83
Smoking history	12.5 ± 10.7	N/A	*N/A*	N/A
Fagerström test	4.1 ± 2.4	N/A	*N/A*	N/A
OCSS	23.8 ± 5.8	N/A	*N/A*	N/A
BIS‐11	69 ± 10.8	58 ± 8.8	3.34	**.002**
STAI‐R	26.5 ± 9.3	21 ± 11.5	1.67	.1
Beck	21.1 ± 14.2	2.75 ± 11.8	1.36	.18
PANAS+	28.35 ± 5.8	29.75 ± 7.5	0.66	.51
PANAS−	20.35 ± 8.7	18.25 ± 8.2	0.78	.44

Abbreviations: Beck, Beck's Depression Questionnaire; BIS‐11, Barratt Impulsiveness Scale‐11 items; OCSS, Obsessive Compulsive Smoking Scale; PANAS+ and PANAS−, Positive Affect and Negative Affect Scale; STAI‐R, State Trait Anxiety Inventory.

### 3.3 Behavioural performance

Smokers did not differ from controls in their response time (*F*_1,38_ < 1, *p* = 0.87), which was not influenced by the type (*F*_2,37_ = 1.3, *p* = 0.27) or the visual perspective of the pictures (*F*_1,38_ = 2.8, *p* = 0.10). Error rate (percentage of false alarms and omissions across trials) was very low and did not differ between smokers (3.9% and 0.7 respectively) and controls (4.5% and 1.2 respectively), *χ* ^2^ (1, *N* = 40) = 2.14, *p* = 0.14.

### 3.4 ERPs reveal an electrophysiological signature of smoking habits

#### 3.4.1 Picture onset

##### P100 component

**FIGURE 2:**
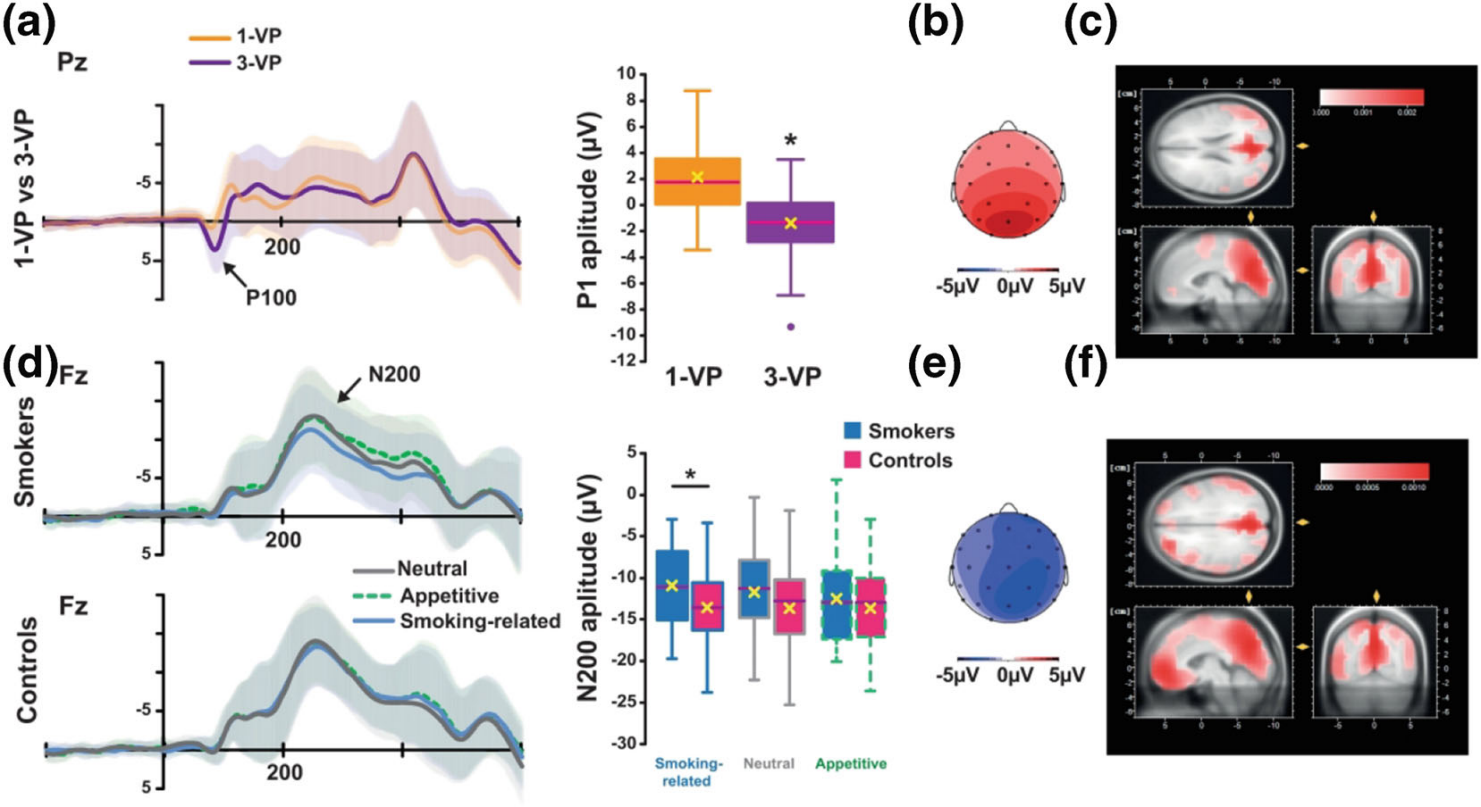
The N200 reflects selective attention capture by drug‐paired cues in smokers. Top panel. ERPs, and especially the P100, evoked in Pz by pictures from a first‐ (light purple) vs. third‐person (dark purple) visual perspective (VP) (a) are represented alongside the P100 mean electrical voltage (μV) over the scalp (b) and its source estimation (LORETA) (c). The P100 evoked by 3‐VP pictures were more negative than those evoked by 1‐VP pictures. Bottom panel. ERPs, and especially the N200, evoked in Fz by neutral, appetitive and smoking‐related pictures in smokers and controls (d) are represented alongside the N200 mean electrical voltage (μV) in central electrodes (e1) (e) and its source estimation (LORETA) (f). Smokers evoked a less negative N200 than controls when presented with a smoking‐related picture. No differences were observed between smokers and controls in the N200 evoked by neutral and appetitive pictures. Note that source estimations of both P100 and N200 effects point to similar putative sources in cortical areas (BA 31 and 7, respectively). *p < .05.

The amplitude of the picture onset‐related P100 (80–110 ms) did not differ between smokers and controls (main effect of group: *F*_1,38_ = 0.10, *p* = 0.75, group × visual perspective interaction: *F*_1,38_ = 3.51, *p* = 0.07, picture type × group interaction: *F*_1,38_ = 2.36, *p* = 0.11, picture type × visual perspective × group interaction: *F*_2,37_ = 0.51, *p* = 0.95). However, pictures presented from first‐person visual perspective evoked a more positive P100 than pictures from a third‐person visual perspective (*F*_1,38_ = 177.60, *p* < 0.0001, _p_η^2^ = 0.82) irrespective of the picture type (main effect of picture type: *F*_2,37_ = 0.69, *p* = 0.51, and visual perspective × picture type interaction: *F*_2,37_ = 0.24, *p* = 0.79) (Figure 2a–d).

##### Posterior N2 component

In contrast, the effect of the visual perspective of the picture on the amplitude of the posterior N2 (240–300 ms) was determined by the electrode (*F*_2,37_ = 11.68, *p* = 0.0001, _p_η^2^ = 0.39), being greater in E1 than E2 and E3 (follow‐up ANOVA in 1‐VP: *F*_2,76_ = 145.81, *p* < 0.0001, _p_η^2^ = 0.84, post hoc comparisons: E1 vs. E2: *t*_2356_ = 22.05, *p* ≤ 0.0001, E1 vs. E3: *t*_2356_ = 24.67, *p* = 0.0001, E2 vs. E3: *t*_2356_ = 2.62, *p* = 0.01; follow‐up ANOVA in 3‐VP: *F*_2,76_ = 128.62, *p* < 0.0001, _p_η^2^ = 0.87, post hoc comparisons: E1 vs. E2: *t*_2356_ = 23.62, *p* ≤ 0.0001, E1 vs. E3: *t*_2356_ = 25.32, *p* = 0.0001, E2 vs. E3: *t*_2356_ = 1.69, *p* = 0.10) and dependent on the group (main effect of group: *F*_1,38_ = 0.67, *p* = 0.42, group × visual perspective interaction: *F*_1,38_ = 3.20, *p* = 0.08, and group × electrode × visual perspective interaction: *F*_2,76_ = 3.25, *p* = 0.04, _p_η^2^ = 0.13) as well as the valence of the picture (main effect of picture type: *F*_2,76_ = 1.74, *p* = 0.18, picture type × visual perspective interaction: *F*_2,76_ = 4.06, *p* = 0.02, _p_η^2^ = 0.19, and picture type × electrode × visual perspective interaction: *F*_4,152_ = 3.48, *p* < 0.01, _p_η^2^ = 0.24), resulting in a four‐way interaction (*F*_4,35_ = 3.13, *p* = 0.03, _p_η^2^ = 0.26). Between‐group post hoc comparisons revealed that the posterior N2 was less negative in smokers than in controls only for smoking‐related 3‐VP pictures (E1: *t*_46_ = 1.98, *p* ≤ 0.05; E2: *t*_46_ = 1.39, *p* = 0.25; E3: *t*_46_ = 1.22, *p* = 0.23; all other *p*s > 0.10) (Figure 2e–h). Additionally, within‐group post hoc comparisons revealed that smoking‐related pictures evoked a less negative N2 at the central electrode than neutral and appetitive pictures in smokers only (Table 3).

**TABLE 3:** Results of the post‐hoc comparisons performed on the N2 amplitudes in smokers as a function of picture type (appetitive vs. smoking‐related vs. neutral), visual perspective (first‐ vs. third‐person) and electrode (E1 vs. E2 vs. E3).

Electrode	Visual perspective	Comparison	*T* value	*P* value
E1	First‐person	Appetitive ‐ Smoking	−4.7	<.0001
		Appetitive ‐ Neutral	0.22	.83
		Smoking ‐ Neutral	4.92	<.0001
	Third‐person	Appetitive ‐ Smoking	−3.77	.0005
		Appetitive ‐ Neutral	−3.54	.0008
		Smoking ‐ Neutral	0.23	.82
E2	First‐person	Appetitive ‐ Smoking	−2.8	.01
		Appetitive ‐ Neutral	0.36	.72
		Smoking ‐ Neutral	3.16	.005
	Third‐person	Appetitive ‐ Smoking	−2.8	.05
		Appetitive ‐ Neutral	−2.32	.05
		Smoking ‐ Neutral	−0.04	.97
E3	First‐person	Appetitive ‐ Smoking	−3.68	<.0001
		Appetitive ‐ Neutral	−0.05	.96
		Smoking ‐ Neutral	3.63	<.0001
	Third‐person	Appetitive ‐ Smoking	−2.84	.01
		Appetitive ‐ Neutral	−2.07	.08
		Smoking ‐ Neutral	0.77	.44

*Note*: *t* and *p* values are reported.

#### 3.4.2 Go/NoGo cue onset

##### Anterior N2‐Go/NoGo component (250–300 ms)

**FIGURE 3:**
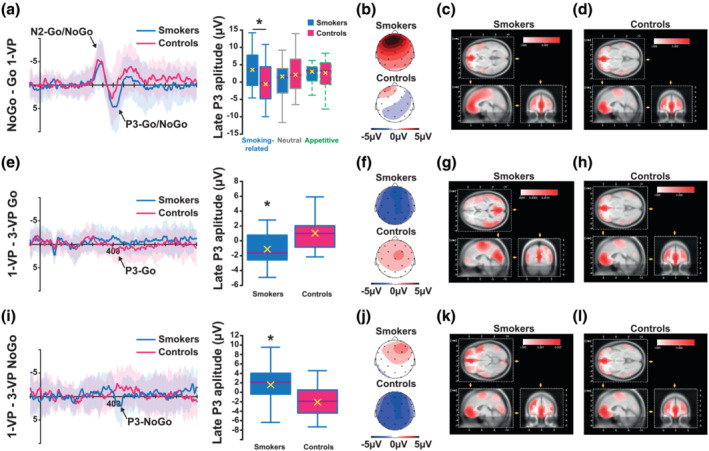
A late P3 selectively tracks the smoking‐related cues presented from a first‐person visual perspective in smokers. (a) The late P3 (420–470 ms) evoked at the F4 electrode following Go or NoGo cues differed between smokers and controls when presented with smoking‐related 1‐VP pictures. The electrical map (b) and source estimation in smokers (c) and controls (d) suggested that the P3 may be associated with alterations in frontal modulations in both smokers and controls (BA 10 and 32). (e) Subsequent analysis of the residual ERP evoked by smoking‐related pictures in Go trials only revealed a differential impact of the visual perspective (the 1‐VP minus 3‐VP) in smokers as compared with controls. The electrical map (f) and the source estimation in smokers (g) and controls (h) suggested that this difference may potentially be due to alterations in modulations of Brodmann area (BA) 23 and 10 in smokers and controls, respectively. (i) In contrast, the residual ERP evoked by smoking‐related pictures presented in NoGo trials revealed a reverse visual perspective effect (1‐VP minus 3‐VP) in each group compared with Go trials. The electrical map (j) and the source estimation in smokers (k) and controls (l) suggested that the P3‐NoGo may present a frontal distribution (BA 10 and 11) in both smokers and controls. *p < .05.

Analysis of *the anterior N2‐Go*/NoGo component (250–300 ms) (Figure 3a) confirmed that the NoGo cue evoked a more negative anterior N2 than the Go cue (*F*_1,38_ = 62.99, *p* < 0.0001, _p_η^2^ = 0.62) while revealing that 1‐VP pictures evoked a more negative anterior N2 than 3‐VP pictures (*F*_1,38_ = 4.88, *p* < 0.05, _p_η^2^ = 0.11). These effects seemed to be independent of one another or of the picture type and of the experimental group since no other main effect or interaction was statistically significant.

##### P3‐Go/NoGo component (310–370 ms and 420–470 ms)

Analysis of the P3 component revealed a complex, group‐dependent, time course of the onset of P3, which occurred later in smokers when presented with smoking‐related pictures. Consequently, the P3 was analyzed over two different time windows, from 310 to 370 ms and from 420 to 470 ms after Go/NoGo cue onset.

The amplitude of the early P3 that was smaller for 1‐VP than 3‐VP pictures (*F*_1,38_ = 6.09, *p* < 0.02, _p_η^2^ = 0.14) was influenced by the type of picture and the regions (picture type × region interaction: *F*_12,27_ = 2.90, *p* = 0.01, _p_η^2^ = 0.56, main effect of picture type: *F*_2,37_ = 1.73, *p* = 0.19, visual perspective × picture type: *F*_2,37_ = 0.16, *p* = 0.85, visual perspective × region: *F*_6,33_ = 5.33, *p* < 0.001, _p_η^2^ = 0.49, visual perspective × picture type × region interaction: *F*_12,27_ = 0.81, *p* = 0.64). Post hoc comparisons revealed that this was due to a smaller P3 evoked by appetitive pictures than neutral pictures in the frontocentral region only (*t*_9538_ = 3.39, *p* = 0.001) and a smaller P3 evoked by smoking‐related pictures than neutral pictures in the frontocentral (*t*_9538_ = 4.80, *p* < 0.001), frontal right (*t*_9538_ = 2.48, *p* = 0.04), frontal left (*t*_9538_ = 3.5, *p* = 0.001) and central regions (*t*_9538_ = 3.36, *p* = 0.002). The interaction between picture type and region was not dependent on having a smoking habit (main effect of group: *F*_1,38_ = 0.36, *p* = 0.55 and group × picture type × region: *F*_12,27_ = 1.37, *p* = 0.24).

The amplitude of the early P3 was also predicated on an interaction between the type of picture and Go/NoGo cue (*F*_2,37_ = 3.42, *p* = 0.04, _p_η^2^ = 0.15). Post hoc comparisons revealed that smoking‐related and appetitive pictures evoked less positive P3 amplitudes than neutral pictures after the NoGo (*t*_9538_ = 9.03, *p* < 0.001 and *t*_9538_ = 5.66, *p* < 0.001, respectively) but not after the Go cue (all *p*s > 0.10). In addition, the P3‐NoGo evoked by the smoking‐related pictures were less positive than those evoked by appetitive pictures (*t*_9538_ = 3.37, *p* = 0.001). This effect was not a characteristic of a smoking habit since it was independent of the group (main effect of group: *F*_1,38_ = 0.36, *p* = 0.55, and group × picture type × cue interaction: *F*_2,37_ = 1.15, *p* = 0.33).

The analysis of the late P3, in contrast, revealed a smoking‐habit‐specific electrophysiological signature. First, the late P3 amplitude was shown to be more positive in the NoGo than the Go condition (*F*_1,38_ = 4.43, *p* = 0.04, _p_η^2^ = 0.10), an effect that varied across regions (main effect of region: *F*_6,33_ = 11.78, *p* < 0.0001, _p_η^2^ = 0.68, and cue × region interaction: *F*_6,33_ = 5.42, *p* < 0.001, _p_η^2^ = 0.50), and as a function of the picture type (main effect of picture type: *F*_2,37_ = 0.38, *p* = 0.69, and cue × region × picture type interaction: *F*_12,27_ = 2.66, *p* = 0.02, _p_η^2^ = 0.54). Thus, the Go/NoGo effect was stronger for smoking‐related pictures than neutral pictures in the frontocentral region (*t*_4750_ = 2.33, *p* = 0.06), whereas it was stronger for appetitive pictures than for neutral pictures in the frontal right (*t*_4750_ = 2.58, *p* = 0.03), central (*t*_4750_ = 3.33, *p* = 0.02) and parietal regions (*t*_4750_ = 2.80, *p* = 0.01).

Finally, the Go/NoGo effect on the late P3 was dependent on whether individuals had a smoking habit, the type and visual perspective of the pictures (group × cue × picture type × visual perspective interaction: *F*_2,37_ = 4.63, *p* = 0.01, _p_η^2^ = 0.20) (Figure 3). Post hoc comparisons revealed that the Go/NoGo effect was stronger for smokers than for controls only for smoking‐related 1‐VP pictures (*t*_42_ = 2.80, *p* = 0.008, all other *p*s > 20) (Figure 3a).

In order to further characterize this effect, the amplitude of the late P3 evoked during Go and NoGo trials was analysed separately. This revealed that the Go/NoGo cue effect was due to an influence of the visual perspective on the late P3‐NoGo (*F*_1,38_ = 6.04, *p* = 0.02, _p_η^2^ = 0.14) and the late P3‐Go (*F*_1,38_ = 7.05, *p* = 0.01, _p_η^2^ = 0.16), both evoked differentially by smokers and controls following presentation of smoking‐related pictures. Post hoc comparisons confirmed that smoking‐related 1‐VP pictures evoked a smaller P3‐Go (*t*_1558_ = 6.56, *p* < 0.0001) than smoking‐related 3‐VP pictures in smokers but a more positive late P3‐Go (*t*_1558_ = 2.38, *p* = 0.02) in controls (Figure 3e–h). Conversely, in NoGo trials, smokers produced a marginally more positive P3‐NoGo (*t*_1558_ = 1.82, *p* = 0.07) with 1‐VP as compared to 3‐VP images, while controls produced a less positive late P3‐NoGo (*t*_1558_ = 9.33, *p* < 0.0001) in the presence of smoking‐related pictures presented from a 1‐VP as compared to a 3‐VP (Figure 3i–l).

#### 3.4.3 Response‐locked potentials

**FIGURE 4:**
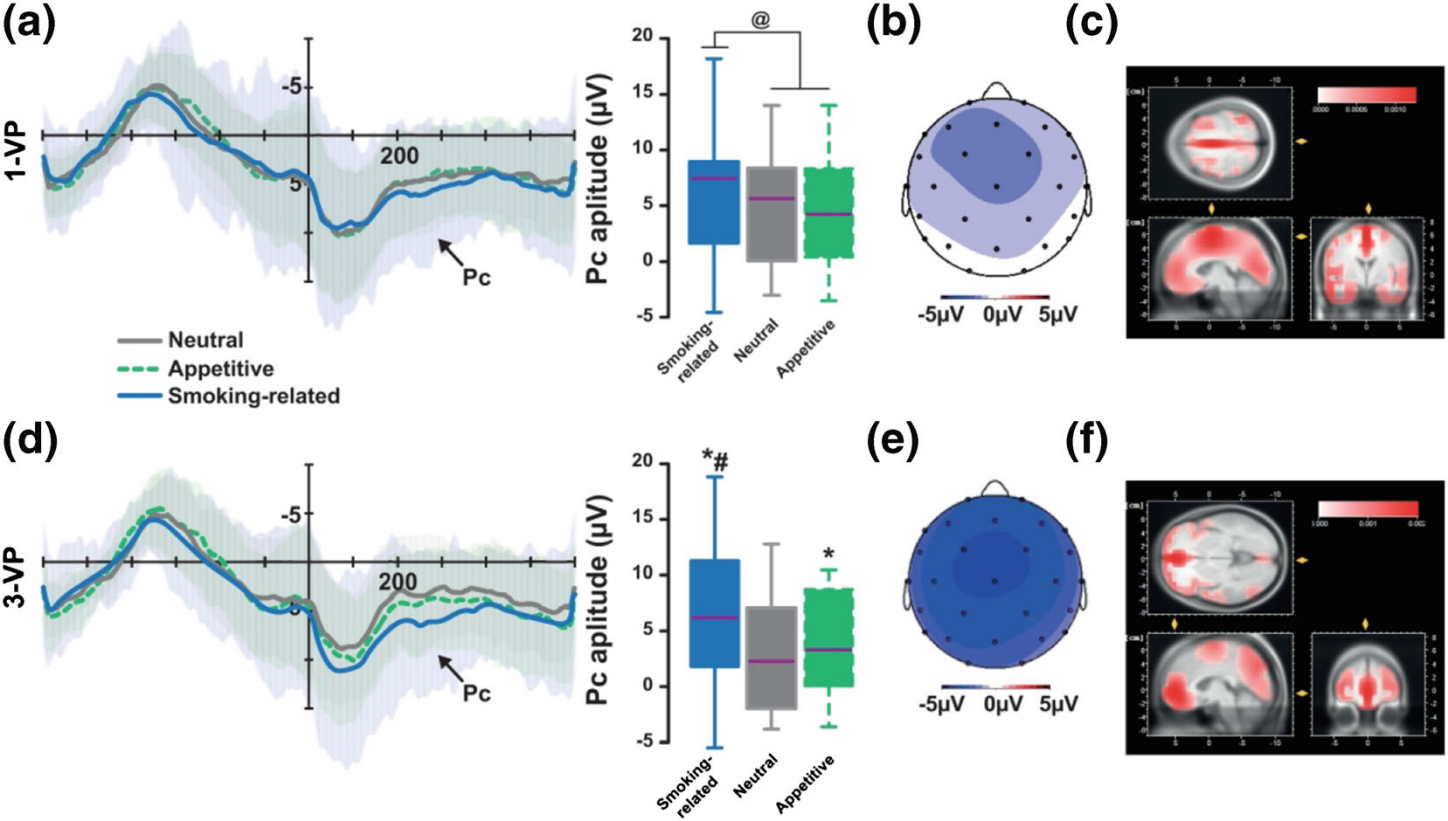
The motor response‐locked Pc in Go trials is greater in smokers for smoking‐related pictures than for appetitive and neutral pictures. (a–c) The amplitude of the Pc (in Cz) evoked by smokers tracked only the difference between responses made with smoking‐related 1‐VP pictures and those made with appetitive and neutral pictures. The latter were associated with a response that evoked a similar Pc with a lower amplitude than that evoked by the former. Source estimation suggested that these effects may potentially be related to modulations of the frontal lobe (BA 6) (b and c). (d) The Pc evoked by smokers following a response made with smoking‐related 3‐VP pictures, which was of a greater amplitude than that evoked by the same pictures from a first‐person visual perspective (1‐VP), was also greater than that evoked by responses made with appetite and neutral pictures. However, when presented from a 3‐VP, appetitive pictures were associated with responses evoking greater Pc than neutral pictures. Source estimation suggested that these effects may be related to modulations of the orbitofrontal cortex (BA 10) (e and f). ^@^ *p* < .05; *different from neural, *p* < 0.05; ^#^different from appetitive, *p* < .05.

Having established an ERP‐based signature of smoking habits on inhibitory mechanisms, we investigated the RRPs in smokers and controls over the 220–300 ms window that followed correct responses in the Go condition. The Pc amplitude was influenced by whether individuals had a smoking habit, the type and the visual perspective of the picture (group × picture type interaction: *F*_2,37_ = 3.46, *p* = 0.04, _p_η^2^ = 0.16, and group × picture type × visual perspective interaction: *F*_2,37_ = 4.19, *p* = 0.02, _p_η^2^ = 0.18). Post hoc comparisons revealed that this was due to a less negative Pc evoked by smokers following a Go response for smoking‐related pictures as compared with neutral (*t*_2356_ = 10.76, *p* < 0.0001) or appetitive pictures (*t*_2356_ = 12.29, *p* < 0.0001, all other *p*s > 0.13) (Figure 4). In addition, the visual perspective of the picture influenced this effect in smokers (*F*_2,38_ = 3.61, *p* = 0.04, _p_η^2^ = 0.16) but not in controls (*F*_2,38_ = 1.53, *p* = 0.23), thereby revealing an important role of the visual perspective in the integration the motivational value of the response in individuals with a smoking habit. Thus, in smokers, responses for smoking‐related 1‐VP pictures evoked a larger Pc than for both neutral (*t*_2356_ = 5.36, *p* < 0.0001) and appetitive pictures (*t*_2356_ = 5.61, *p* < 0.0001), which did not differ from each other (*t*_2356_ = 0.25, *p* = 0.81). This effect could be attributed to the modulation of the frontal cortex (Brodmann area, BA 6) by source estimation (Figure 4a–d). In contrast, responses made for smoking‐related 3‐VP pictures evoked a more positive Pc than for smoking‐related 1‐VP pictures (*t*_2356_ = 2.63, *p* = 0.01). This larger smoking picture‐related response‐evoked Pc is also greater than that evoked for neutral (*t*_2356_ = 14.43, *p* < 0.0001) and appetitive pictures (*t*_2356_ = 11.11, *p* < 0.0001). However, responses made for appetitive 1‐VP images evoked a larger Pc than those made for neutral pictures (*t*_2356_ = 3.32, *p* < 0.001) (Figure 4e–h). Source estimation indicated that these effects may all be related to modulations of the orbitofrontal cortex (BA 10).

## 4 DISCUSSION

Smokers performed as well as controls overall in the modified Go/NoGo task developed for this study, the two groups made very few errors and showed fast response times, both well within the standard range reported for this type of task (Buzzell et al., 2014; Evans et al., 2009). Thus, smokers involved in the present study, while having a smoking habit, did not display a global impairment of their inhibitory system, in agreement with previous studies (Buzzell et al., 2014; Evans et al., 2009). Differences in experimental design may explain why our current findings deviate from previous reports of poorer inhibition (i.e., lower accuracy) in No‐Go trials only, in smokers with similar Fagerström scores (Luijten et al., 2011). Unlike previous studies, we included non‐smoking‐related appetitive image controls, incorporated first‐person visual perspective and altered the trial presentation rate. Behavioural measures such as accuracy are also quite volatile and may not always capture inhibitory control deficits.

Performance in the task was associated with classical anterior N2‐Go/NoGo and P3‐Go/NoGo ERP components, the magnitude of which was predicated on the nature of the trial (Go vs. NoGo cue), as previously described (Kok et al., 2004; Randall & Smith, 2011), but not influenced by having a smoking habit. Drug‐cue processing has also been related to a modulation of the LPP, a positive component usually recorded in centroparietal regions between 300 and 600 ms after stimulus onset (Littel & Franken, 2007; Minnix et al., 2013; Robinson et al., 2016). The differential modulation of the LPP by drug‐related cues as compared with drug‐unrelated cues has long been suggested to represent differential cognitive processing of the emotional or motivational signification of the stimulus (Littel & Franken, 2007; Minnix et al., 2013; Robinson et al., 2016). For example, Versace et al. (2011) reported LPP of greater positive amplitudes when evoked by smoking‐related, pleasant and unpleasant stimuli than those evoked by neutral stimuli. We expected to detect the motivational component of the response to cue presentation in the range of the LPP, that is, in the P3 (310–370 ms), taking the Go/NoGo cue as stimulus onset (Agudelo‐Orjuela et al., 2021). As anticipated, smoking‐related and appetitive pictures evoked a less positive P3 (310–370 ms, that is 560 ms after the onset of the pictures) than neutral pictures in both smokers and controls, thereby demonstrating that the two groups processed the emotional characteristics of the pictures similarly. These results seem at odds with previous evidence that smokers elicit smaller N2 and larger P3 than controls (Buzzell et al., 2014; Liu et al., 2019; Mobascher et al., 2010). These apparent discrepancies are potentially attributable to major differences in the experimental design of these studies and ours, such as the use of a classic Go/No Go task at high speed or that of an auditory odd‐ball task in which the P300 reflects more attentional rather than inhibitory processes (Kropotov, 2010). To the best of our knowledge, only Luijten et al. (2011) used a design relatively similar to the one deployed here in their study that reported that smokers evoked a less negative anterior N2‐NoGo than controls. This effect, obtain with sample sizes similar to those of the present study, was interpreted as a global reduction of the efficiency of the (early) inhibitory processes due, according to the authors, to high task demands. However, in Luijten et al. (2011), smoking‐related or unrelated pictures were presented simultaneously with the Go/NoGo cue, conditions not amenable properly to investigate the interaction between motivational and inhibitory processes. Thus, Luijten et al. (2011) were unlikely to observe the kind of interaction between the type of picture and the type of Go/NoGo cue on the anterior N2 or P3 components that we revealed in our study in which, appetitive, smoking‐related or neutral pictures were presented 250 ms before the Go/NoGo cue, in order specifically to investigate these interactions (Agudelo‐Orjuela et al., 2021).

In individuals with a SUD, drug‐related cues generate specific multisensory representations associated with overt and covert motivational mechanisms, including approach bias (Watson et al., 2012). Importantly, many smoking‐related cues are response‐produced, and therefore perceived from a first‐person visual perspective (e.g., automatic embodiment, see Canizales et al., 2013; Galang et al., 2020), by smokers, in whom they act as conditioned reinforcers. Conditioned reinforcers not only bridge delays to reinforcement, but they also facilitate the development of incentive habits and the subsequent development of compulsive drug seeking (Belin et al., 2013; Belin & Everitt, 2010; Fouyssac et al., 2022). The specific influence of conditioned reinforcers on motivational processes has been illustrated by the greater influence cues exert on behaviour when presented from first‐ than third‐person visual perspective (Yalachkov et al., 2012). Accordingly, we manipulated the visual perspective of each stimulus, which influenced, in the first instance, the amplitude of the P100 in both smokers and controls. Similar visual perspective effects previously reported have been related to somatosensory processing involved in representations of oneself and others (Rigato et al., 2019). In addition to the general influence the visual perspective exerts on early ERP components, smoking‐related pictures evoked a posterior picture‐locked N2 of smaller amplitude than neutral and appetitive pictures, only when presented from a first‐person visual perspective. This observation is in agreement with the long‐established modulation of early ERP components (e.g., N1 and N2; for a review, see Rangaswamy & Porjesz, 2014) by drug‐related cues in individuals with a SUD, which has been interpreted as a drug‐specific attention bias. Descriptive source estimation suggests these effects may originate respectively from the posterior cingulate cortex (BA 31) and the parietal superior cortex (BA 7), areas that have been related to the processing of the spatial information in goal‐oriented behaviours (Hadjidimitrakis et al., 2019) and associated CS‐induced attention bias (Engelmann et al., 2009).

Beyond the processing of the motivational value of cues, this study also identified the nature of the influence of these cues on inhibition processing. Thus, the emotionally loaded appetitive and smoking‐related pictures evoked a smaller early P3‐NoGo (310–370 ms) than neutral pictures in both smokers and controls, a well‐established signature of the impact of the emotional characteristics of stimuli on inhibition (Agudelo‐Orjuela et al., 2021). This was followed by a smaller late P3‐Go (420–470 ms), in smokers only, evoked by smoking‐related compared with neutral pictures, and only when presented from a first‐person visual perspective. Smoking‐related 1‐VP pictures may, therefore, provoke a transient impairment of inhibitory processes associated with an automatic recruitment of the incentive motivational processes related to the influence of conditioned reinforcers on the expression of incentive habits (Belin et al., 2013; Jones et al., 2018).

However, follow‐up analysis showed that the modulation in smokers of the late P3‐Go/NoGo (420–470 ms) by smoking‐related cues was not only due to a decrease of the P3‐Go amplitude, but also to an increase of that of the P3‐NoGo. Smokers produced a smaller P3‐Go in the presence of smoking‐related 1‐VP as compared with 3‐VP pictures, a profile opposite to that shown by the controls. Source estimation suggests that this effect may potentially be associated with an activation of the posterior cingulate cortex (BA 23) or the prefrontal cortex (BA 10) in smokers and controls, respectively. This decrease in the P3‐Go amplitude shown by smokers may represent the impact of previously activated incentive habit‐related Stimulus–Response rules by smoking‐related 1‐VP cues (i.e. attention bias, posterior N2 effect at picture onset), resulting in the facilitation of response processing when both the drug‐related and task‐related cues point to or converge towards a similar response (Detandt et al., 2017; Watson et al., 2012; Wiers et al., 2013). Since controls have never learnt about, or indeed experienced response‐produced smoking‐related cues, it would be expected that they did not show such facilitation of response processing by these stimuli when presented from a first‐person visual perspective, which to some extent may even be alien to them. It was therefore not surprising that controls displayed the exact opposite neurophysiological signature to that shown by smokers, for example, controls evoked a larger P3‐Go than smokers when presented with a smoking‐related 1‐VP picture, suggesting that such an unfamiliar situation requires more response processing than that associated with appetitive images.

The specific inhibition processing profile of smoking habits was associated with a unique response potential signature. The analysis of the response potentials in Go trials revealed that smokers evoked a smaller, less negative response potential than controls for responses associated with a smoking‐related picture as compared with neutral or appetitive pictures, irrespective of the visual perspective in which they are presented. The time course of this effect (220 and 300 ms) is in line with the P300‐like correct Positivity component (Pc). Even though the nature of the cognitive function it reflects remains relatively elusive (Bates et al., 2004), the Pc is increasingly considered to reflect a post‐response monitoring process sensitive to both the subjective value of the outcome (Falkenstein et al., 2000) and the congruency between responses in that it tends to decrease when two stimuli call for a similar response (Mathalon et al., 2002). Accordingly, the decrease in the Pc amplitude observed in the present study in smokers when responding following the presentation of a smoking‐related cue suggests a facilitation of the post‐response evaluation processing reflective of the recruitment of ingrained automatic, outcome value independent, responding mediated by stimulus–response associations (Robbins & Costa, 2017). Source estimation suggests that this could potentially be associated with the activation of Sensory‐Motor‐Area (SMA, BA 6) when the response occurs after the presentation of a smoking‐related 1‐VP picture.

While the putative association of the posterior cingulate cortex with the smaller P3‐Go evoked by smokers in response to a smoking‐related 1‐VP picture discussed above may point towards an influence of the perception of any stimulus presented as first‐person visual perspective as being more related to the self and thereby influencing the level of familiarity or affective salience of the image (Murray et al., 2012; Zilverstand et al., 2018), the source estimation in the SMA of the smaller Pc evoked by smokers responding following the presentation of smoking‐related 1‐VP pictures suggests a specific recruitment of automatic responding mediated by an action knowledge network (Yalachkov et al., 2009; Yalachkov & Naumer, 2011). In addition, while only responses made by smokers following a smoking‐related picture evoked a lower Pc than neutral pictures when presented from a first‐person visual perspective, responding following both appetitive and smoking‐related pictures evoked a smaller Pc than following neutral pictures when presented from a third‐person visual perspective, reflective of a facilitation of post‐response evaluation that is proportional to the emotional value of the stimulus when presented from a third‐person visual perspective. In line with this interpretation, the differential Pcs evoked for responses following presentations of stimuli from a third‐person visual perspective did not originate in the SMA, as shown for Pc evoked by responses made following smoking‐related pictures presented from a first‐person visual perspective, but instead in the orbitofrontal cortex (BA 10), which has been related to decision‐making processes based on stimulus–reward mapping (Young & Shapiro, 2011).

### 4.1 Conclusion

While the influence of drug‐, and in particular alcohol‐, related CSs on inhibitory processing in individuals with a SUD has been well documented (for a meta‐analysis, see Jones et al., 2018), that of smoking‐related cues, especially when presented from the same visual perspective as that of the response by which they are produced, in individuals with a smoking habit was less well understood. By comparing the influence of smoking‐related cues presented either from a first‐ or third‐person visual perspective to that of drug‐unrelated appetitive pictures on the neurophysiological correlates of inhibitory processing (Versace et al., 2017), and response monitoring (Falkenstein et al., 2000; Mathalon et al., 2002), this study reveals a selective signature of the engagement of the inhibitory system and automatic response processing by smoking‐related pictures in smokers.

Together the results of this experiment suggest that in individuals with a smoking habit, who have a long history of drug foraging under the control of the conditioned reinforcing properties of response‐produced smoking‐related cues, thence experienced from a first‐person visual perspective, smoking‐related cues presented from the same visual perspective as conditioned reinforcers engage weak inhibitory processing while facilitating the recruitment of habitual responding.

## AUTHOR CONTRIBUTIONS

Julien Dampuré and David Belin designed the experiments. Julien Dampuré conducted the experiments and the analysis of the data. Julien Dampuré and David Belin wrote the manuscript. Horacio A. Barber contributed to the design of the study and provided intellectual input. Paola Agudelo‐Orjuela and Maartje Van Der Meij contributed to the experiments.

## CONFLICT OF INTEREST STATEMENT

The authors have no conflict of interest to declare.

### PEER REVIEW

The peer review history for this article is available at https://publons.com/publon/10.1111/ejn.15942.

## Data Availability

The data can be available at https://docs.google.com/spreadsheets/d/1VyYDZckcA6%2Dz%5FCRX7YnK98MfoW%5FOX7Dd/edit%3Fusp%3Dsharing%26ouid%3D113770177571978399708%26rtpof%3Dtrue%26sd%3Dtrue.
